# The Anthrax Toxin Receptor 1 (ANTXR1) Is Enriched in Pancreatic Cancer Stem Cells Derived from Primary Tumor Cultures

**DOI:** 10.1155/2019/1378639

**Published:** 2019-05-02

**Authors:** Sonia Alcalá, Paola Martinelli, Patrick C. Hermann, Christopher Heeschen, Bruno Sainz

**Affiliations:** ^1^Department of Biochemistry, Universidad Autónoma de Madrid (UAM), Madrid, Spain; ^2^Department of Cancer Biology, Instituto de Investigaciones Biomédicas “Alberto Sols” (IIBM), CSIC-UAM, Madrid, Spain; ^3^Chronic Diseases and Cancer Area 3, Instituto Ramón y Cajal de Investigación Sanitaria (IRYCIS), Madrid, Spain; ^4^Institute for Cancer Research, Comprehensive Cancer Center, Medical University of Vienna, Vienna, Austria; ^5^Department of Internal Medicine I, Ulm University, Germany; ^6^School of Medical Sciences, University of New South Wales Sydney, Australia

## Abstract

Pancreatic ductal adenocarcinoma (PDAC) is currently the fourth leading cause of cancer-related mortality. Cancer stem cells (CSCs) have been shown to be the drivers of pancreatic tumor growth, metastasis, and chemoresistance, but our understanding of these cells is still limited by our inability to efficiently identify and isolate them. While a number of markers capable of identifying pancreatic CSCs (PaCSCs) have been discovered since 2007, there is no doubt that more markers are still needed. The anthrax toxin receptor 1 (ANTXR1) was identified as a functional biomarker of triple-negative breast CSCs, and PDAC patients stratified based on *ANTXR1* expression levels showed increased mortality and enrichment of pathways known to be necessary for CSC biology, including TGF-*β*, NOTCH, Wnt/*β*-catenin, and IL-6/JAK/STAT3 signaling and epithelial to mesenchymal transition, suggesting that ANTXR1 may represent a putative PaCSC marker. In this study, we show that ANTXR1^+^ cells are not only detectable across a panel of 7 PDAC patient-derived xenograft primary cultures but ANTXR1 expression significantly increased in CSC-enriched 3D sphere cultures. Importantly, ANTXR1^+^ cells also coexpressed other known PaCSC markers such as CD44, CD133, and autofluorescence, and ANTXR1^+^ cells displayed enhanced CSC functional and molecular properties, including increased self-renewal and expression of pluripotency-associated genes, compared to ANTXR1^−^ cells. Thus, this study validates ANTXR1 as a new PaCSC marker and we propose its use in identifying CSCs in this tumor type and its exploitation in the development of CSC-targeted therapies for PDAC.

## 1. Introduction

While pancreatic ductal adenocarcinoma (PDAC) currently represents the fourth most frequent cause of cancer-related deaths worldwide, it is expected to surpass both colorectal cancer and lung cancer to become the second leading cause of cancer-related deaths by 2030 [1]. These alarming statistics are due to several key factors, including the lack of early and specific symptoms that impedes timely detection and diagnosis. As such, approximately 80% of patients present with nonresectable advanced metastatic disease [2]. Likewise, the intrinsically high metastatic nature and resistance of PDAC tumors to chemotherapy and radiation [2] make treatment efforts essentially ineffective, resulting in a median overall survival rate of less than 7 months and a 5-year survival rate of less than 7% [1]. These inherent and defining PDAC characteristics are believed to be due in part to the existence of a subpopulation of stem-like cells within the tumor known as cancer stem cells (CSCs), which drive tumorigenesis, metastasis, and chemoresistance [3–5].

In an effort to isolate and better understand the biological properties of these rare stem-like cells, which have been identified across diverse solid tumors including brain, ovarian, breast, colon, and liver cancers [6–9], researchers have focused on identifying markers present on their cell surface. In the context of pancreatic CSCs (PaCSCs), we and others have made significant strides in identifying markers that are capable of identifying PaCSCs in cell lines [10, 11] and primary cultures established from patient-derived xenografts (PDXs) [12–16], directly from primary patient PDAC tumors [14, 16], and in blood [17]. Nonetheless, appreciating that there likely exist many subpopulations of PaCSCs, or hierarchies within specific PaCSC populations, it is still necessary to continue to search for additional PaCSC markers.

The anthrax toxin receptor 1 (ANTXR1) is a 564 amino acid transmembrane protein encoded by the tumor endothelial marker 8 (*TEM8*) gene [18] and is one of the three receptors known to facilitate the entry of anthrax toxin into cells [19]. ANTXR1 (or TEM8) specifically binds the nontoxic protective antigen (PA) component of anthrax toxin [20]. In the context of cancer, ANTXR1/TEM8 was initially shown by St. Croix et al. to be overexpressed on the tumor vasculature and to play a role in tumor angiogenesis [18]; however, in 2001, a study by Duesbery et al. demonstrated that *in vitro* treatment of V12 H-ras-transformed NIH 3T3 cells or *in vivo* injection of anthrax lethal toxin ((LeTx): PA plus lethal factor (LF)) into athymic nude mice implanted with ras-transformed cells led to a strong antitumor response and, in some cases, caused complete tumor regression of xenografts [21]. This study strongly suggested that in addition to being expressed on the tumor vasculature [18], ANTXR1 is also expressed on tumor cells allowing for LeTx-mediated elimination of these cells. It would later be confirmed that ANTXR1 is expressed on cancer cells of different tumor entities including breast, neuroblastoma, and melanoma [22–24], and neuroblastoma and melanoma xenografts are also sensitive to LeTx [23], highlighting the broad role that this protein may play in cancer cell biology. It is now appreciated that ANTXR1 or TEM8 can also bind the cleaved C5A fragment of collagen alpha 3 (VI) [25, 26] and interact with the Wnt signaling lipoprotein receptor-related protein 6 (LRP6) [26], modulating collagen cleavage [26] or downstream Wnt signaling [26, 27], respectively, pathways important in the development of the tumor microenvironment or in cancer cell “stemness.” It would not be until 2013, however, that a study by Chen et al. would show for the first time that ANTXR1 is expressed on metastatic breast CSCs and functions in collagen signaling, as well as Wnt signaling, ZEB1 expression, and CSC self-renewal, invasion, tumorigenicity, and metastasis [26]. While this highlights an important role for ANTXR1 in breast CSCs, the expression of ANTXR1 on CSCs from other tumor entities, such as PDAC, has not been explored to date.

To bridge this gap, we used 3D sphere cultures established from PDXs and enriched in PDAC CSCs to evaluate the expression of ANTXR1 on PaCSCs alone or in combination with other known CSC markers, such as CD133, CD44, or autofluorescence. We show not only that ANTXR1 is enriched in PaCSC sphere cultures and coexpresses with other known PaCSC markers but that ANTXR1-positive cells have increased self-renewal capacity and an overall higher expression of pluripotency-associated genes compared to ANTXR1-negative cells. As such, these studies validate ANTXR1 as a new PaCSC marker and we further propose its use in identifying CSCs in PDAC and its exploitation in the development of CSC-targeted therapies.

## 2. Materials and Methods

### 2.1. Gene Expression Datasets and GSEA Analyses

In addition to the TCGA gene expression dataset, the dataset from Janky et al. [28] and that from Moffitt et al. [29] were downloaded from GEO (GSE62165 and GSE71729); the dataset from Jandaghi et al. [30] was downloaded from ArrayExpress (E-MTAB-1791); the dataset from Bailey et al. was included in a supplementary figure of their published work [31], and the META dataset, containing datasets GSE15471, GSE16515, GSE22780, and GSE32688, was generated as described in [32]. Patients were stratified based on *ANTRX1* expression using the optimal cutoff calculated through http://www.kmplot.com [33], and survival analysis was performed with R. Log-rank test was used to determine statistical significance. Cox regression was used to calculate the hazard ratio. The samples included in the two groups were compared in GSEA, using the Hallmark gene set collection of the MSigDB database. The GSEA module of the GenePattern suite from the Broad Institute was used, with 1000 permutations, and FDR < 25% was considered statistically significant.

### 2.2. Primary Human Pancreatic Cancer Cells and Macrophage-Conditioned Media

PDAC patient-derived xenografts (PDXs) were obtained under a Material Transfer Agreement with the Spanish National Cancer Centre (CNIO), Madrid, Spain (reference no. I409181220BSMH). Xenografts were processed as previously described [34] to establish low-passage primary PDAC PDX-derived *in vitro* cultures. PDAC PDX-derived cultures are referred to by a random number designation (e.g., Panc185, Panc215, Panc253, Panc286, PancB06, PancB023, and Panc354). PDAC PDX-derived cultures and L3.6pl cells were maintained in RPMI media supplemented with 10% FBS and 50 units/ml penicillin/streptomycin and fungizone (all from Thermo Fisher Scientific). All cultures were tested for mycoplasma at least every 4 weeks, and microsatellite analysis was performed to authenticate all cell lines used. Macrophage-conditioned media were generated from MCSF-treated blood monocyte-derived cultures from one healthy donor as previously described [16].

### 2.3. Western Blot

For the analysis of TEM8 and Tubulin protein levels, cultures were lysed with RIPA buffer (Sigma) containing a protease inhibitor cocktail (Roche Applied Science, Indianapolis, IN). Protein (50 *μ*g) was resolved by SDS-PAGE and transferred to PVDF membranes (Amersham Pharmacia, Piscataway, NJ). Membranes were then blocked with blocking buffer (1x TBS; 5% BSA (*w*/*v*); 0.5% Tween 20 (*v*/*v*)), incubated with a 1 : 500 dilution of rabbit anti-TEM8 (cat no. ab21270, Abcam) or a 1 : 5000 dilution of mouse anti-Tubulin (cat no. T9026, Sigma) overnight at 4°C, washed 5 times with wash buffer (1x PBS; 0.5% Tween 20 (*v*/*v*)), incubated with horseradish peroxidase-conjugated goat anti-rabbit or goat anti-mouse antibody (Amersham), and washed again to remove unbound antibodies. Bound antibody complexes were detected with SuperSignal chemiluminescent substrate (Amersham).

### 2.4. Flow Cytometry

Cells were analyzed with a 4-laser Attune NxT Acoustic Cytometer (Thermo Fisher Scientific). Samples were resuspended in FLOW buffer (1x PBS; 3 mM EDTA (*v*/*v*); 3% FBS (*v*/*v*)), and the following fluorescently tagged antibodies were used to label cells for 30 minutes at 4°C: mouse monoclonal anti-human CD133-PE (1 : 20, cat no. 130-110-962, Miltenyi), mouse monoclonal anti-human CD44-PE (1 : 20, cat no. 130-095-180, Miltenyi), and mouse monoclonal anti-human EpCAM-APC (1 : 20, cat no. 130-113-260, Miltenyi). For ANTXR1 detection, cells were first labeled with rabbit anti-human TEM8 (1 : 50, cat no. ab21270, Abcam), washed three times with 1x PBS and subsequently incubated with goat-anti-rabbit (Alexa 647 1 : 500, cat no. A27040, Invitrogen). DAPI was used to mark and exclude dead cells, and data were analyzed using the software FlowJo v9.3 (Tree Star Inc., Ashland, OR). Autofluorescent cells were excited with blue laser 488 nm and selected as the intersection with the filters 530/40 and 580/30 as previously described [14].

### 2.5. Sphere Formation Assay

Spheres were generated by culturing 2 × 10^3^ PDAC cells per ml in ultralow attachment plates (Corning, New York, NY) in suspension using serum-free DMEM/F12 supplemented with B27 (1 : 50, Invitrogen, Waltham, MA), 20 ng/ml bFGF, and 50 units/ml penicillin/streptomycin for a total of 7 days, allowing spheres to reach a size of >75 *μ*m, as previously described [35]. To quantify spheres of >40 *μ*m, 1 ml of sample volume was analyzed with a CASY cell counter (Roche Applied Sciences, Mannheim, Germany). The CASY cell counter measuring principle is based on a capillary particle counter with pulse area analysis that permits the determination of cell count, cell concentration, cell volume (peak), and average cell diameter, specifically diameters of 40-80 *μ*m, 80-120 *μ*m, and >120 *μ*m.

### 2.6. RNA Preparation and Real-Time Quantitative PCR (RTqPCR)

The GTC method [36] was used to isolate total RNA. Briefly, 1 *μ*g of purified RNA was used for cDNA synthesis using the Maxima First Strand cDNA Synthesis Kit for RT-qPCR with dsDNase (Thermo Fisher Scientific), followed by SYBR green (PowerUp™ SYBR™ Green Master Mix, Thermo Fisher Scientific) RTqPCR using an Applied Biosystems StepOnePlus™ real-time thermocycler (Thermo Fisher Scientific). The thermal cycling conditions used consisted of the following: a predenaturation cycle (10 min at 95°C) followed by 40 cycles of denaturation (15 sec at 95°C) and annealing/extension (1 min at 60°C). To determine relative mRNA copy numbers, standard curves comprised of serial dilutions of plasmids containing the target coding sequences were included. Cycle thresholds were normalized to *β*-actin levels. Primers for the following human transcripts were used. *KLF4*: forward, 5′-ACCCACACAGGTGAGAAACC-3′ and reverse, 5′-ATGTGTAAGGCGAGGTGGTC-3′; *SOX2*: forward, 5′-AGAACCCCAAGATGCACAAC-3′ and reverse, 5′-CGGGGCCGGTATTTATAATC-3′; *OCT3/4*: forward, 5′-CTTGCTGCAGAAGTGGGTGGAGGAA-3′ and reverse, 5′-CTGCAGTGTGGGTTTCGGGCA-3′; *NANOG*: forward, 5′-TGAACCTCAGCTACAAACAGGTG-3′ and reverse, 5′-AACTGCATGCAGGACTGCAGAG-3′; *ANTXR1/TEM8*: forward, 5′-ACAGGGTCCTCTGCAGCTTCAA-3′ and reverse, 5′-GTCAGAACAGTGTGTGGTGGTGAT-3′; and *β*-actin: forward, 5′-GCGAGCACAGAGCCTCGCCTT-3′ and reverse, 5′-CATCATCCATGGTGAGCTGGCGG-3′.

### 2.7. Statistical Analyses

Results are presented as means ± standard error of the mean (sem) unless stated otherwise. Statistical analysis was performed using two-tailed Student's *t*-test or one-tailed Fisher's test, and significance was considered for *p* < 0.05. All analyses were performed using GraphPad Prism version 5.0c (San Diego, California, USA). Additional experimental details can be found in the Supplementary Materials and Methods.

## 3. Results

### 3.1. *ANTXR1/TEM8* Is Overexpressed in PDAC

**Figure 1:**
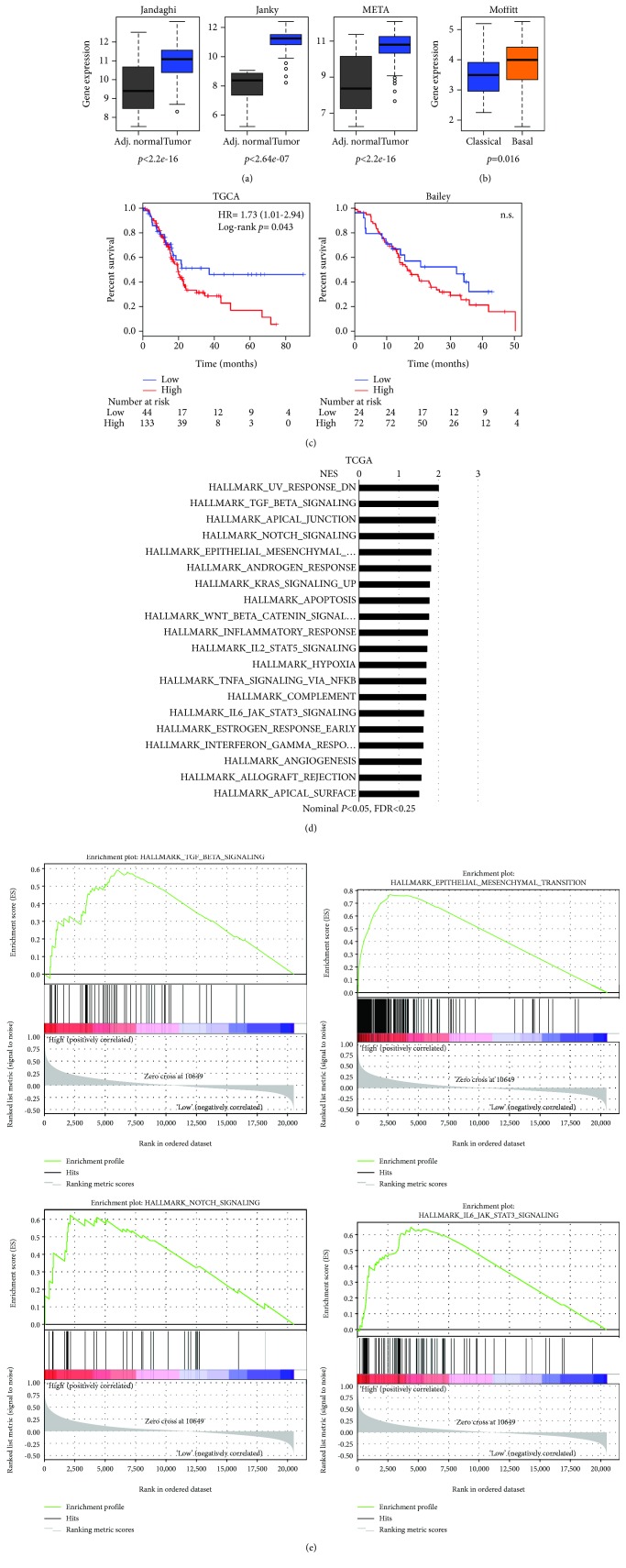
*ANTXR1/TEM8* is overexpressed in PDAC. (a) Boxplots showing the differential expression of *ANTXR1* in PDAC samples versus normal adjacent tissue in three independent series of transcriptomics data (Jandaghi: *n* = 13 adj. normal, *n* = 118 tumor; Janky: *n* = 70 adj. normal, *n* = 108 tumor; META: *n* = 13 adj. normal, *n* = 118 tumor). (b) Boxplots showing the differential expression of *ANTXR1* in classical versus basal tumors from the Moffitt series (*n* = 89 classical, *n* = 36 basal). (c) Kaplan-Meier curves showing the overall survival of PDAC patients, stratified based on high and low *ANTRX1* expression using the optimal cutoff calculated through http://www.kmplot.com for the TCGA and Bailey et al. datasets. The hazard ratio (HR) and number of patients at risk are shown. (d, e) Gene sets enriched in the transcriptional profiles of tumors belonging to the top *ANTXR1* high-expression group, compared with the bottom-expression group in the TCGA dataset. A nominal *p* value of <0.05, FDR < 25% is considered statistically significant. Shown are the NES (normalized enrichment score) values for each pathway using the Hallmark gene sets (d) and example enrichment plots for TGF-beta, EMT, NOTCH, and IL-6/JAK6/STAT3 signaling (e).

While *ANTXR1/TEM8* overexpression has been observed in different tumor entities [22, 23], its expression and putative role in PDAC have not been rigorously examined to date. Using the publicly available transcriptome datasets (Jandaghi et al. [30], Janky et al. [28], META dataset [32], and Moffitt et al. [29]), the transcriptional levels of ANTXR1 expression were evaluated. We observed that *ANTXR1* mRNA expression was significantly elevated in whole pancreatic tumor samples versus adjacent normal tissue (Figure 1(a)) and tumors of the basal subtype (Figure 1(b)), having worse prognosis, expressed significantly higher levels of *ANTXR1* compared to classical subtype tumors. For the TCGA dataset and the Bailey et al. series [31], well-annotated clinical data is available and was used to determine if high levels of *ANTXR1* expression correlated with decreased overall survival. As expected, a clear deviation and significant decrease in median overall survival of *ANTXR1* high-expressing patients compared to the *ANTXR1* low-expressing patients were observed in the TCGA dataset, and although not significant (*p* = 0.92), a similar trend was observed with the Bailey et al. series (Figure 1(c)). Next, we performed GSEA comparing the patient samples expressing high levels of ANTXR1 (the top 75%) to those expressing low levels of *ANTXR1* (bottom 25%) from the TCGA and Bailey et al. datasets. Using the “Hallmark” gene set collection, we observed significantly and commonly enriched pathways across both series, including TGF-*β*, NOTCH, Wnt/*β*-catenin, and IL-6/JAK/STAT3 signaling and epithelial to mesenchymal transition (EMT) gene enrichment (Figures 1(d) and 1(e) and Supplementary Figure S1A-B). Likewise, using the “Kegg” gene set collection, enrichment of similar pathways, such as Wnt/*β*-catenin, EMT, and TGF-*β* signaling, was observed in both datasets (Supplementary Figure S1C-D). Moreover, enrichment in pathways associated with collagen remodeling, extracellular matrix, and adhesion were also enriched, consistent with findings associating ANTXR1 with cleaved C5A collagen binding (Supplementary Figure S1C-D) [25, 26].

### 3.2. ANTXR1/TEM8 Is Overexpressed in Pancreatic CSCs

**Figure 2:**
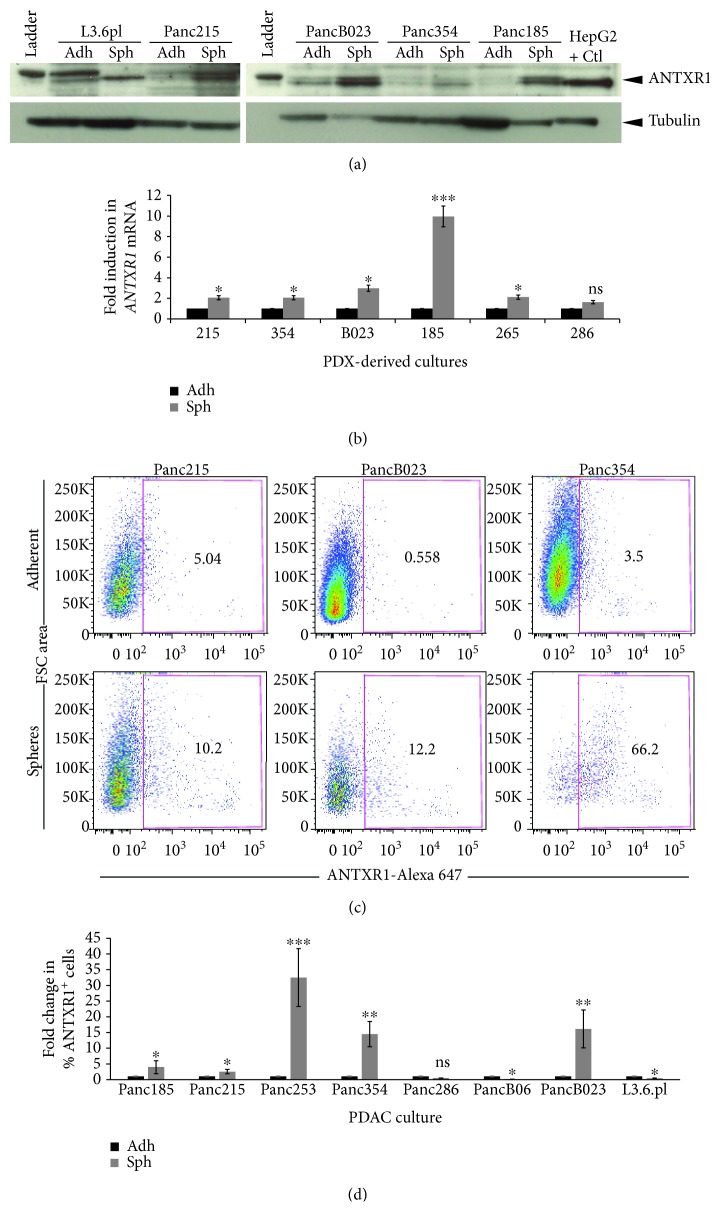
ANTXR1/TEM8 is overexpressed in pancreatic CSCs. (a) Expression of TEM8 detected by Western blotting, in total lysates from L3.6pl or the indicated PDX-derived cell lines cultured in adherence (Adh) or as sphere (Sph). Tubulin was used as a loading control. Total HepG2 cell lysate was used a positive control (+ Ctl). (b) RTqPCR analysis of *ANTXR1* relative mRNA expression levels in L3.6pl or the indicated PDX-derived cell lines cultured in adherence (Adh) or as sphere (Sph). mRNA expression levels for each target gene are normalized to *β*-actin levels (*n* = 4 samples per group). Fold changes were calculated compared to Adh (set at 1.0). (c) Representative flow cytometric analysis of TEM8 staining in Panc215, PancB023, or Panc354 cells cultured in adherence (Adh) or as sphere (Sph). Shown are the percent-positive cells present within the single-cell live debris-free population. (d) Histogram summarizing the percent of ANTXR1-postive cells present in L3.6pl or the indicated PDX-derived cell lines cultured in adherence (Adh) or as sphere (Sph). Fold changes were calculated compared to Adh (set at 1.0) (triplicate samples and *n* = 3 − 4 experiments per cell line). ^∗^ *p* < 0.05, ^∗∗^ *p* < 0.01, and ^∗∗∗^ *p* < 0.001.

Since many pathways involved in CSC biology were enriched in the *ANTXR1* high population and considering the previously published association between ANTXR1 expression and breast CSCs [26], we set out to determine if ANTXR1 is overexpressed in PaCSCs. Using L3.6pl cells and a panel of PDX-derived primary cultures, ANTXR1 protein and mRNA expression was determined in adherent cultures (non-CSC enriched) and in 3D sphere cultures (CSC enriched) by Western blot and RTqPCR analysis, respectively (Figures 2(a) and 2(b)). Using both approaches, we observed increased expression of ANTXR1 in CSC-enriched sphere cultures versus adherent monolayer controls. This increase was not only evident at the total protein and mRNA level, but ANTXR1 cell surface expression was also significantly increased in CSC-enriched sphere cultures (Figure 2(c)) across 5 out of 7 PDX cultures tested (Figure 2(d)). Interestingly, although L3.6pl cells have been shown to contain a CD133 and CXCR4 double-postive CSC population [13], these cells did not show an increase in ANTXR1 cell surface expression upon culturing as 3D spheres.

### 3.3. ANTXR1/TEM8-Positive Cells Are Detectable in Tumors and Can Be Induced

**Figure 3:**
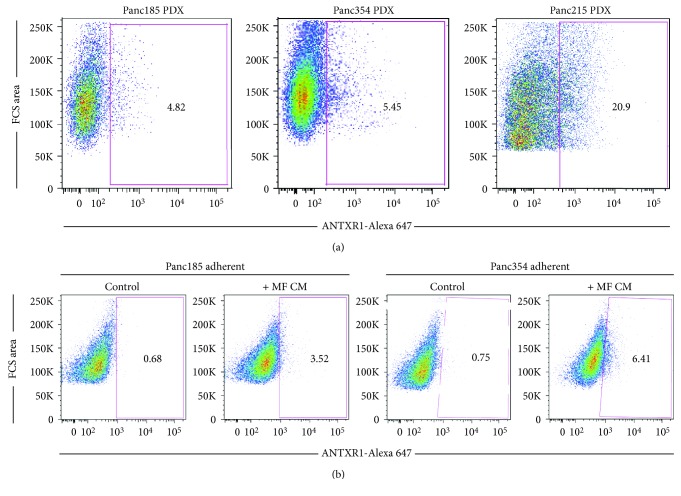
ANTXR1/TEM8-positive cells are detectable in freshy digested PDXs and can be induced. (a) Representative flow cytometric analysis of TEM8 staining in digested Panc185, Panc354, or Panc215 PDX tumors. Shown are the percent-positive cells present within the single-cell, live, debris-free, and EpCAM^+^ population (*n* = 1 experiment). (b) Representative flow cytometric analysis of TEM8 staining in Panc185 or Panc354 cells cultured in adherence and treated with control media or MF CM for 48 hours. Shown are the percent-positive cells present within the single-cell live debris-free population (*n* = 2 experiments).

To determine if ANTXR1^+^ cells could be detected in freshly digested PDXs, three low-passage PDXs were digested and the percentage of ANTXR1^+^ cells was determined within the live (DAPI-negative) human epithelial cell (EpCAM-positive) population. As shown in Figure 3(a), ANTXR1^+^ cells were detectable in all three PDXs tested, with Panc215 PDX containing the highest percentage. These data would indicate that ANTXR1 expression is not a consequence of *in vitro* culture, but rather, EpCAM^+^/ANTXR1^+^ cells are present within the tumor.

We previously published that the CSC compartment can be activated and induced to expand upon treatment with macrophage- (MF-) conditioned media (CM) from monocyte-derived macrophages polarized to an M2 phenotype with MCSF [16]. To determine whether ANTXR1^+^ cells can also be induced to expand, Panc185 and Panc354 adherent monolayer cultures were treated with MF CM from M2-polarized macrophages and flow cytometry analysis was performed to detect the percentage of ANTXR1 cells following 48 hours of treatment. As expected, in both primary cultures tested, the percentage of ANTXR^+^ cells increased by 5.2- and 8.5-fold, respectively, (Figure 3(b)).

### 3.4. ANTXR1/TEM8 Is Coexpressed with the Pancreatic CSC Markers CD133, CD44, and Autofluorescence

**Figure 4:**
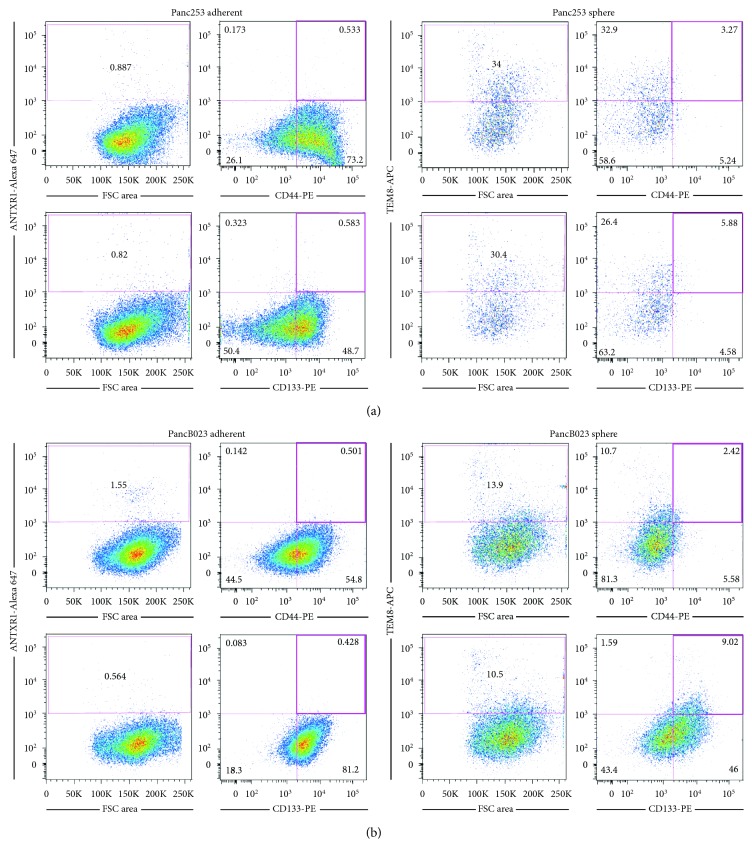
ANTXR1/TEM8 is coexpressed with pancreatic CSC markers CD133 and CD44. Representative flow cytometric analysis of TEM8, TEM8 and CD44, or TEM8 and CD133 staining in (a) Panc253 or (b) PancB023 cells cultured in adherence or as spheres. Shown are the percent-positive cells present within the single-cell, live, debris-free, and EpCAM^+^ population (*n* = 2 experiments).

**Figure 5:**
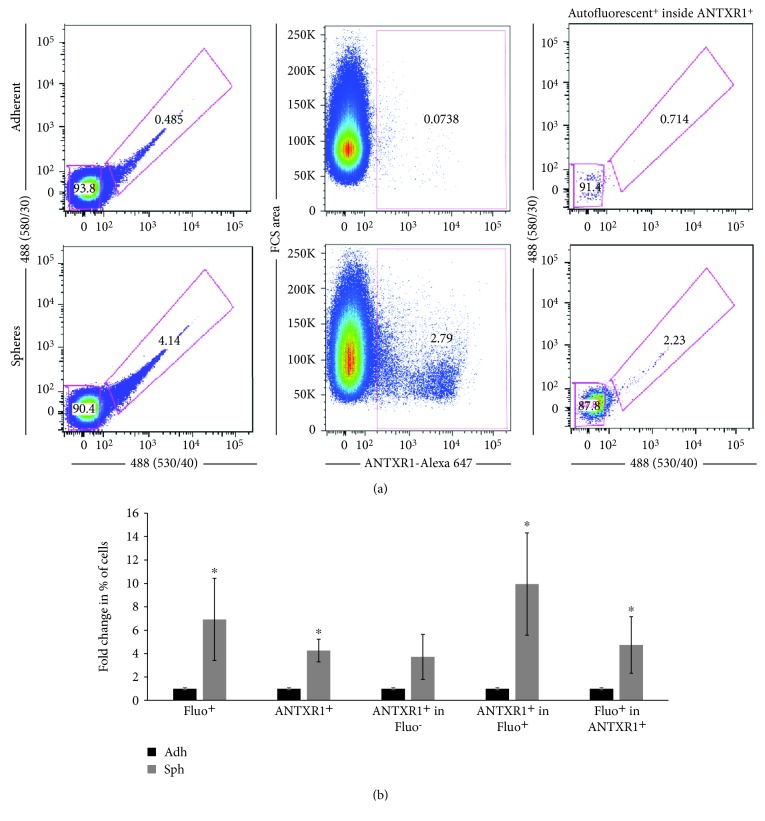
ANTXR1/TEM8 is coexpressed with the pancreatic CSC marker autofluorescence. (a) Gating strategies for the detection of autofluorescent-positive cells, ANTXR1-positive cells, and autofluorescent-positive cells within the ANTXR1-positive population in adherent or sphere cultures. (b) Histogram summarizing the percent of autofluorescent-positive or ANTXR1-positive cells, ANTXR1-positive cells in the autofluorescent-negative or positive populations, or the percent of autofluorescent-positive cells in the ANTXR1-positive population in adherent or sphere cultures. Fold changes were calculated compared to Adh (set at 1.0) (triplicate samples and *n* = 3 − 4 experiments). ^∗^ *p* < 0.05.

We reasoned that if ANTXR1 identifies PaCSCs, these cells should also coexpress other known CSC markers, such as CD44 or CD133. To determine whether ANTXR1-postive cells coexpress CD44 or CD133 together with ANTXR1, Panc253 and PancB023 cells were analyzed by flow cytometry. While less than 1% of adherent monolayer cultures were CD44^+^/ANTXR1^+^ or CD133^+^/ANTXR1^+^ double-positive, in sphere conditions, not only did ANTXR1 cell surface expression increase but the CD44^+^/ANTXR1^+^ and CD133^+^/ANTXR1^+^ double-positive populations increased to 3.27% and 5.88%, respectively, in Panc253 and to 2.42% and 9.02%, respectively, in PancB023 cells, indicating that ANTXR1^+^ cells also express known CSC markers (Figure 4). The fact that only a fraction of the ANTXR^+^ cells coexpressed CD133 or CD44 was not surprising as we have shown that hierarchies exist within CSC populations [14]. Finally, using autofluorescence, the recently discovered CSC marker that is the result of riboflavin accumulation in ABCG2-coated intracellular vesicles exclusively present in epithelial CSCs [14], we assessed whether autofluorescent CSCs express ANTXR1. Similar to CD133 and CD44, we could detect autofluorescent-positive (Fluo^+^) cells within the ANTXR1^+^ population and this population increased when CSCs were enriched in 3D sphere cultures (Figures 5(a) and 5(b)). Likewise, within the Fluo^+^ population, a significant percentage of cells were also ANTXR^+^ (Figure 5(b)).

### 3.5. ANTXR1/TEM8-Positive PDAC Cells Possess CSC Functional and Molecular Traits

**Figure 6:**
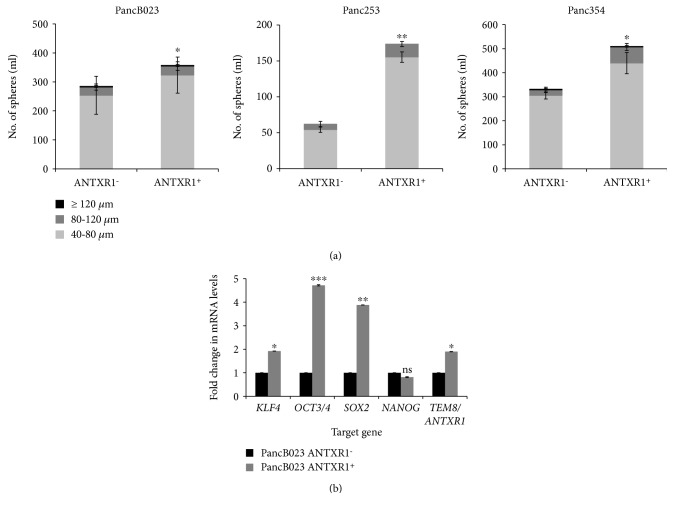
ANTXR1/TEM8-positive PDAC cells possess CSC functional and molecular traits. (a) Sphere-forming capacity of ANTXR1^+^ and ANTXR^−^ cells sorted from PancB023, Panc253, and Panc354 cells following seven days in non-adherent culture conditions. Shown is the total number of spheres determined/1 ml categorized by size in *μ*M (triplicate samples and *n* = 2 experiments). (b) RTqPCR analysis of relative mRNA expression levels for the indicated genes in ANTXR1^+^ and ANTXR1^−^ cells sorted from PancB023. mRNA expression levels for each target gene are normalized to *β*-actin levels. Fold changes were calculated compared to ANTXR1^−^ (set at 1.0) (triplicate samples and *n* = 2 experiments).

Finally, to evaluate whether ANTXR1^+^ cells share functional traits described for other CSC populations [4], ANTXR1^+^ cells were isolated from PancB023, Panc253, and Panc354 via FACS. The self-renewal capacity of ANTXR1^−^ and ANTXR1^+^ cells was assessed in a sphere formation assay. In line with increased self-renewal, ANTXR1^+^ cells isolated from all three PDX-derived cell lines formed significantly more spheres than ANTXR1^−^ cells (Figure 6(a)). CSCs have also been shown to have increased expression of pluripotency-associated transcripts, which is necessary for the maintenance of the CSC state. In ANTXR1-postive-sorted PancB023 cells, *KLF4*, *OCT3/4*, and *SOX2* expression was significantly higher compared to ANTXR1-negative-sorted cells. *NANOG* mRNA levels were not significantly modulated, and as expected, *ANTXR1/TEM8* mRNA levels were 2-fold higher in ANTXR1-positive-sorted cells (Figure 6(b)). The sum of these data supports the claim that ANTXR1^+^ cells possess functional and molecular traits characteristic of CSCs.

## 4. Discussion

The need for markers that specifically identify CSCs is driven by the need to target and or eliminate these highly tumorigenic and chemoresistant cells. Unfortunately, to date, no universal marker that identifies CSCs across all tumor types exists. This is likely due to the fact that different CSC clones are present within a tumor, CSCs are a state rather than an entity, and hierarchies exist within CSC subpopulations [5]. Thus, as we increase the number of markers available to identify and isolate these cells, we will increase our understanding of their biology and role in cancer. In this study, we build upon the observation that ANTXR1 is expressed on CSCs by showing for the first time its expression on PaCSCs. Prior to this work, Chen et al. identified ANTXR1 as a functional biomarker of normal stem cells and breast cancer stem-like cells [26]; however, the presence of ANTXR1 on CSCs of other tumor entities was lacking. We show that ANTXR1^+^ cells are present in PDX-derived cultures from 7 PDAC patients and its expression significantly increases when cells are cultured as 3D spheres. Since growth in anchorage-independent serum-free conditions promotes the apoptosis of differentiated cells and the enrichment of CSCs, the observed and significant increase in ANTXR1 expression on sphere-derived cells strongly suggested a CSC link. The latter was confirmed by showing that ANTXR^+^ cells coexpress the known CSC markers CD44 and CD133 [13], as well as the recently discovered CSC marker autofluorescence [14]. Interestingly, when the highly aggressive L3.6pl cell line was cultured as 3D spheres, no increase in ANTXR1 cell surface expression was observed. L3.6pl cells were derived from parental FG cells by multiple *in vivo* passaging to select for highly metastatic cells [37], which have been shown to contain CD133^+^/CXCR4^+^ metastatic CSCs [13]. The low levels of ANTXR1 expression on L3.6pl spheres may suggest that ANTXR1^+^ CSCs were lost during *in vivo* selection of this cell line or that ANTXR1^+^ cells are not enriched in cell lines that contain primarily metastatic PaCSC subpopulations [13]. Indeed, we have shown that hierarchies do exist within CSC populations [14] and CSC markers are not always equally enriched for across different conditions (e.g., sphere formation, tumor formation, or chemoresistance). For example, using the CSC biomarker autofluorescence, we observed that not all Fluo^+^CSCs express CD133 nor are all CD133^+^ cells autofluorescent. Along those lines, not all ANTXR1^+^ cells were positive for CD133, CD44, or autofluorescence and vice versa. Therefore, the use of ANTXR1 in combination with other CSC markers may be identifying a distinct CSC subpopulation, with a potentially different biological role. At the functional and molecular level, however, ANTXR1^+^ cells showed significantly higher self-renewal capacity and increased expression of pluripotency-associated genes compared to their ANTXR1^−^ counterparts. Thus, ANTXR1^+^ cells satisfy two main CSC requirements, but more studies are necessary to fully understand the stem-like state of ANTXR1^+^ cells. For example, *in vivo* assessment of the tumorigenic and metastatic capacity of these cells is still needed.

It is important to note that ANTXR1 is not the only anthrax receptor [19]. In addition to ANTXR1/TEM8, the capillary morphogenesis gene 2 (CMG2)/ANTXR2 can also interact with LeTx. These two receptors, however, are biologically and functionally different. For example, TEM8 has been shown to play a role in the regulation of tubule formation and endothelial cell migration, while CMG2 may be more specific to endothelial cell proliferation. In addition, TEM8 and CMG2 appear to differentially bind collagen I and collagen VI, respectively, or collagen IV and laminin, respectively, [38]; however, CMG2 was recently shown to also act as a receptor for collagen VI and mediate its intracellular degradation [39], indicating that TEM8 and CMG2 may have some overlapping functions. In the context of cancer, contradictory roles for CMG2 have been described. For example, while CMG2 was shown to regulate prostate cancer cell adhesion and invasiveness [40], in breast cancer, CMG2 inhibited breast cancer cell growth and was inversely correlated with disease progression and prognosis [41]. Interestingly, a recent 2018 publication by Ji et al. has shown for the first time that CMG2 is not only expressed on gastric cancer stem-like cell but is necessary to maintain the stem-like phenotype of these cells via Wnt/*β*-catenin pathway activation [42]. It appears that Wnt/*β*-catenin signaling may be one of the main signaling pathway downstream of the anthrax receptor(s) in CSCs. Interestingly, we also observed that in patients with high *ANTXR1* expression, Wnt/*β*-catenin signaling was enriched; however, more studies are necessary to determine whether ANTXR1 present on the cell surface of PaCSCs maintains “stemness” via Wnt/*β*-catenin signaling.

Finally, the facts that LeTx binds to ANTXR1, LeTx can reduce tumor growth *in vivo*, and ANTXR1 is expressed/enriched on the surface of CSCs suggest that the antitumor effects observed with LeTx in the studies published by Duesbery et al. [21] and Rouleau et al. [23] were likely a consequence of CSC targeting. These studies highlight the very real possibility that ANTXR1 could be utilized to target CSCs *in vivo* as a means of treating cancer. Indeed, targeting the anthrax receptors for the purposes of inhibiting tumor angiogenesis and tumor growth has been extensively investigated (reviewed in [38]) and the effects of LeTx and modified forms of PA administered along with LF have shown strong and encouraging antiangiogenic and antitumorigenic effects in preclinical cancer models. Likewise, adapting PA and LF components to increase their biological activity and minimize toxicity, synthesizing mutant PA moieties with more potent antiangiogenic and tumor growth properties [43], and developing anti-ANTXR1 antibodies have also been explored. Regarding the latter, NCI researchers and Novartis were the first to develop potent ANTXR1-specific mAbs using phage display libraries and to test the anticancer efficacy of these mAbs in preclinical xenograft mouse models of colon cancer and in a syngeneic mouse model of melanoma [44]. The authors showed that antibodies developed against the TEM8 extracellular domain not only had broad antitumor activity but could also be used in combination with anticancer chemotherapeutics without added toxicity. Since this study was published in 2012, MMAE-linked anti-ANTXR1 antibody drug conjugate (ADC) treatments have been developed and shown to be well tolerated and capable of inducing tumor regression or tumor eradication in multiple solid tumor types, inhibiting metastatic growth and prolonging overall *in vivo* survival [45]. Likewise, CAR T cell-based immunotherapy targeting ANTXR1/TEM8 has been developed and shown to have cytotoxic specificity for tumor endothelial cells as well as ANTXR1-positive triple-negative breast cancer cells [46] or gastric adenocarcinoma cells [47]. Thus, in light of our findings showing that PaCSCs express ANTXR1, the aforementioned ANTXR1-based therapies should be tested in preclinical models of pancreatic cancer.

In summary, we show in this study that ANTXR1 efficiently identifies PaCSCs, ANTXR1^+^ cells coexpress other known PaCSC markers such as CD44, CD133, and autofluorescence, and ANTXR1^+^ cells display enhanced CSC functional and molecular properties. Thus, we propose that ANTXR1 should be added to the list of PaCSC markers and utilized for the development of future anti-PaCSC-based therapies. Finally, considering the aforementioned CMG2 findings in gastric cancer stem-like cells, it is not unlikely that CMG2 may also be expressed on PaCSCs and the use of both ANTXR1 and ANTXR2 may further facilitate the capacity to enrich for/isolate a purer CSC population. Experiments towards this end are underway. Without a doubt, the role of anthrax toxin receptors appears to extend beyond that of merely interacting with LeTx. Their roles in tumor vasculature, in tumor angiogenesis, and now in tumor CSC biology highlight the dynamic roles that these proteins play in multiple biological processes. Time will tell in what other processes they participate in, but for now, their utility in cancer should be fully exploited.

## Data Availability

The data used to support the findings of this study are available from the corresponding author upon request.

## Supplementary Materials

Supplementary Materials Figure S1: ANTXR1/TEM8 is overexpressed in PDAC.
