# Supplementary material for: The Anthrax Toxin Receptor 1 (ANTXR1) Is Enriched in Pancreatic Cancer Stem Cells Derived from Primary Tumor Cultures
Source: Stem Cells Int. 2019 May 2;2019:1378639. doi: 10.1155/2019/1378639 (PMC6525821; doi:10.1155/2019/1378639)
Supplement: Supplementary Materials — Figure S1: ANTXR1/TEM8 is overexpressed in PDAC. [file 1378639.f1.pdf]

Figure S1 – *ANTXR1/TEM8* is overexpressed in PDAC

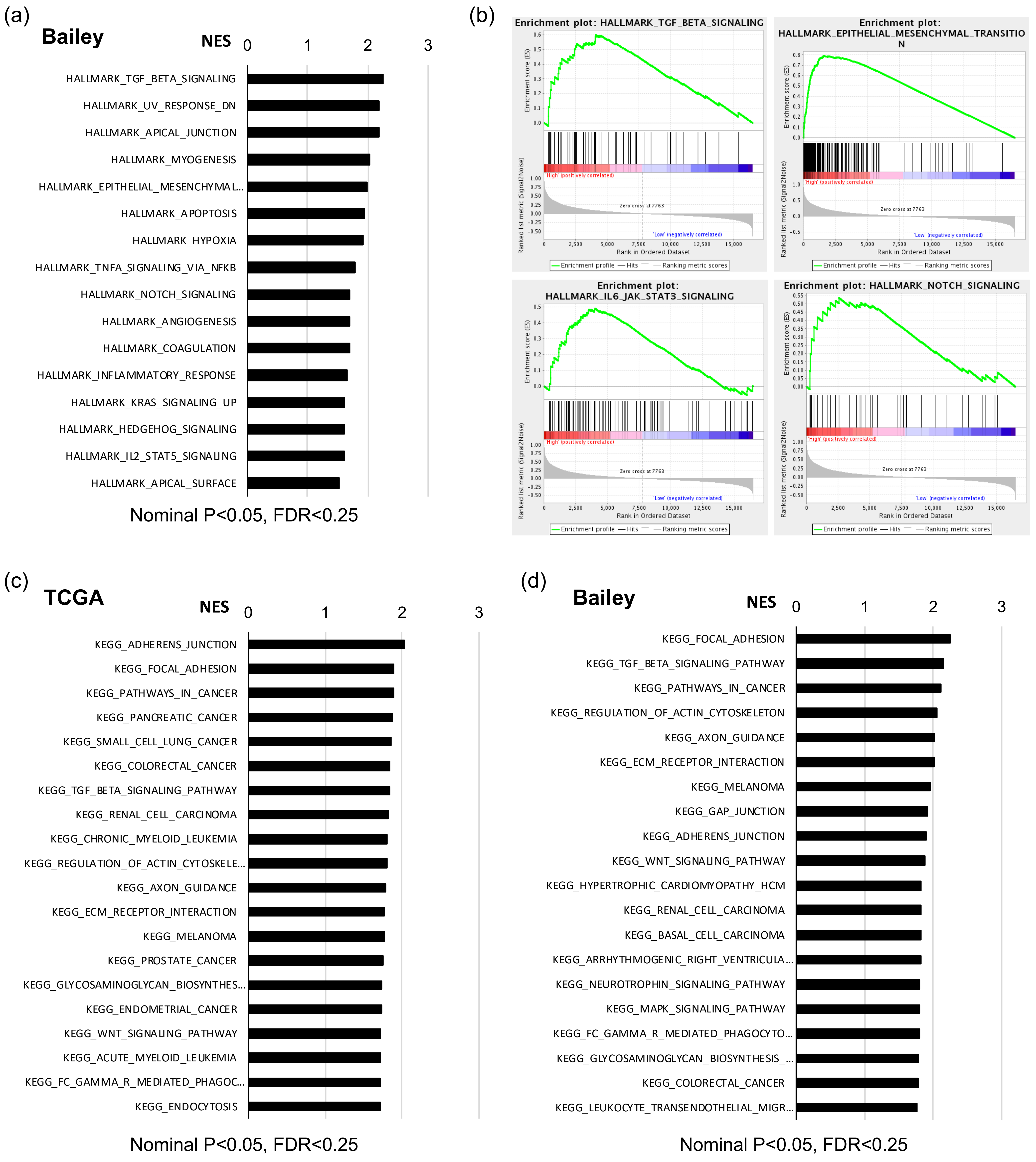

Figure S1. *ANTXR1/TEM8* is overexpressed in PDAC. (a-b) Genesets enriched in the transcriptional profiles of tumors belonging to the top *ANTXR1* high expression group, compared with the bottom expression group in the Bailey *et al.* dataset. A nominal p value of <0.05, FDR<25% is considered statistically significant. Shown are the NES values for each pathway using the Hallmark genesets (a) and example enrichment plots for TGF-beta, EMT, Notch, and IL-6/JAK6/STAT3 signaling (b). (c-d) Kegg genesets enriched in the transcriptional profiles of tumors belonging to the top *ANTXR1* high expression group, compared with the bottom expression group in the (c) TCGA and (d) Bailey *et al.* datasets. A nominal p value of <0.05, FDR<25% is considered statistically significant. Shown are the NES values for each pathway.
